# Prediction of Nephrotoxicity Associated With Cisplatin-Based Chemotherapy in Testicular Cancer Patients

**DOI:** 10.1093/jncics/pkaa032

**Published:** 2020-04-23

**Authors:** Sara L Garcia, Jakob Lauritsen, Zeyu Zhang, Mikkel Bandak, Marlene D Dalgaard, Rikke L Nielsen, Gedske Daugaard, Ramneek Gupta

**Affiliations:** p1 Department of Health Technology, Technical University of Denmark, Kgs. Lyngby, Denmark; p2 Department of Oncology, Copenhagen University Hospital, Copenhagen, Denmark; p3 Key Laboratory of Genetic Network Biology, Institute of Genetics and Developmental Biology, University of Chinese Academy of Sciences, Beijing, China; p4 Sino-Danish Center for Education and Research, Eastern Yanqihu campus, University of Chinese Academy of Sciences, Beijing, China

## Abstract

**Background:**

Cisplatin-based chemotherapy may induce nephrotoxicity. This study presents a random forest predictive model that identifies testicular cancer patients at risk of nephrotoxicity before treatment.

**Methods:**

Clinical data and DNA from saliva samples were collected for 433 patients. These were genotyped on Illumina HumanOmniExpressExome-8 v1.2 (964 193 markers). Clinical and genomics-based random forest models generated a risk score for each individual to develop nephrotoxicity defined as a 20% drop in isotopic glomerular filtration rate during chemotherapy. The area under the receiver operating characteristic curve was the primary measure to evaluate models. Sensitivity, specificity, and positive and negative predictive values were used to discuss model clinical utility.

**Results:**

Of 433 patients assessed in this study, 26.8% developed nephrotoxicity after bleomycin-etoposide-cisplatin treatment. Genomic markers found to be associated with nephrotoxicity were located at *NAT1*, *NAT2*, and the intergenic region of *CNTN6* and *CNTN4*. These, in addition to previously associated markers located at *ERCC1*, *ERCC2*, and *SLC22A2*, were found to improve predictions in a clinical feature–trained random forest model. Using only clinical data for training the model, an area under the receiver operating characteristic curve of 0.635 (95% confidence interval [CI] = 0.629 to 0.640) was obtained. Retraining the classifier by adding genomics markers increased performance to 0.731 (95% CI = 0.726 to 0.736) and 0.692 (95% CI = 0.688 to 0.696) on the holdout set.

**Conclusions:**

A clinical and genomics-based machine learning algorithm improved the ability to identify patients at risk of nephrotoxicity compared with using clinical variables alone. Novel genetics associations with cisplatin-induced nephrotoxicity were found for *NAT1*, *NAT2*, *CNTN6*, and *CNTN4* that require replication in larger studies before application to clinical practice.

Standard treatment in patients with disseminated testicular cancer is chemotherapy consisting of bleomycin-etoposide-cisplatin (BEP). Cisplatin is also central in the treatment of many other solid tumors such as bladder, ovarian, and lung cancer (1). Treatment containing cisplatin has a wide range of side effects, one of which is nephrotoxicity (2,3).

Cisplatin is excreted by the kidneys and may induce nephrotoxicity resulting in glomerular filtration rate (GFR) decline (4). Maintenance of sufficient renal function during treatment with chemotherapy is vital, and identification of patients at risk for developing nephrotoxicity could influence the treatment of choice if alternatives exist. Additionally, impaired renal function has been associated with increased risk of cardiovascular disease (5), which may pose a problem in long-term cancer survivors.

Previous studies have improved the understanding of molecular mechanisms of cisplatin-induced nephrotoxicity (6), and several candidate gene studies have identified single-nucleotide polymorphisms (SNPs) associated with cisplatin-induced nephrotoxicity (7–9). However, these studies were conducted with surrogate measures of GFR (creatinine clearance or estimated GFR) rather than measured GFR as outcome.

The scope of this study was 2-fold: first, to conduct a genome-wide association study (GWAS) using a linear model controlling for cisplatin dosage (high or normal) to identify new genetic variants associated with cisplatin-induced nephrotoxicity; and second, to investigate the utility of germline genetic markers together with clinical prognostic factors to predict nephrotoxicity using a random forest-recursive feature elimination algorithm. Patients treated for disseminated testicular cancer were chosen for this study because this patient group does not normally have comorbidity, which could influence renal function.

## Methods

### Patients

Patients were identified in the Danish Testicular Cancer-Late cohort (10), which includes 2572 Danish patients treated for testicular cancer from 1984 through 2007. Clinical features from 433 patients were originally extracted from hospital files as registered in the Danish Testicular Cancer database (Table 1). In 2014, all patients with measurements of renal function before and after treatment with BEP were invited to deliver a saliva sample for DNA analysis (Supplementary Figure 1, available online). Patients provided informed consent, and the study was approved by the regional ethical committee (H-2-2012-044) and the National Board of Data Protection (2012-41-0751).

**Table 1.** Comparison of baseline characteristics between affected (GFR high-drop) and nonaffected patients ^a^

Characteristics	Affected, No. (%)	Nonaffected, No. (%)	*P* ^b^
No. of patients	116 (26.8)	317 (73.2)	
Clinical characteristics			
Age, median (IQR)	34 (27-43)	30 (26-37)	.001
BEP regimen			
Normal dose	92 (79.3)	295 (93.4)	<.001
Double dose	24 (20.7)	21 (6.6)	
Unknown	—	1	
GFR before treatment, median (IQR), mL/min/1.73 m^2^	128 (115-139)	119 (110-131)	.001
GFR after treatment, median (IQR), mL/min/1.73 m^2^	88 (75-99)	109 (100-119)	<.001
Cisplatin, median (IQR), mg/m^2^	400 (391-410)	400 (300-400)	<.001
Treatment cycles			
3	20 (17.2)	97 (30.6)	<.001
** **4	72 (62.1)	199 (62.8)	
5 or more	6 (5.2)	14 (4.4)	
High dose	18 (15.5)	7 (2.2)	
Histology			
Seminoma	23 (19.8)	68 (21.5)	.78
Nonseminoma	93 (80.2)	249 (78.5)	
Prognostic group			
Good	71 (61.2)	277 (87.4)	<.001
Intermediate	30 (25.9)	35 (11.0)	
Poor	15 (12.9)	5 (1.6)	
Stage			
Extragonadal	15 (12.9)	15 (4.7)	.87
Stage Im	7 (6.0)	30 (9.6)	
Stage Iia	22 (19.1)	80 (25.5)	
Stage Iib	21 (18.1)	77 (24.5)	
Stage Iic	23 (19.8)	42 (13.4)	
Stage III	28 (24.1)	70 (22.3)	
Unknown	—	3	

^a^ BEP = bleomycin-etoposide-cisplatin; GFR = glomerular filtration rate; IQR = interquartile range.

^b^ *P* values were calculated by 2-sided Mann-Whitney *U* test for continuous or ordinal characteristics. For “histology,” *P* value was calculated by χ^2^ test.

### Treatment and Renal Measurement

All 433 patients received 3 cycles or more of BEP. The majority received normal-dose cisplatin 20 mg*/*m^2^ × 5 q3w, etoposide 100 mg/m^2^ × 5 q3w, and bleomycin 15 IU/m^2^ q1w, and 25 patients received double-dose cisplatin and etoposide: cisplatin 40 mg*/*m^2^ × 5 q3w, etoposide 200 mg/m^2^ × 5 q3w, and bleomycin 15 IU/m^2^ q1w. Hydration remained uniform over time with 2 L isotonic saline before cisplatin and an additional 1-2 L after. Diuretics were administered only in special cases, and no magnesium was added to hydration. There was no predefined cutoff of renal function where patients would not receive cisplatin-based triplets; however, to ensure toxicity was related to treatment, only patients with a GFR greater than 90 mL/min/1.73m^2^ before chemotherapy were included.

GFR was measured by the 1-sample 51Cr-ethylenediaminetetra acetic acid clearance technique using 2 samples 200 minutes after tracer injection and normalized to a body surface area (BSA) of 1.73 m^2^.

### Genomic Information

Genomic DNA was collected and purified using GeneFiX Saliva DNA Midi Kit from Isohelix (Harrietsham, UK). DNA samples were prepared at DTU Multi-Assay Core (Lyngby, Denmark) and genotyped at AROS Applied Biotechnology A/S (Aarhus, Denmark) using Illumina HumanOmniExpressExome-8 v1.2 chip (964 193 markers).

Genomic data were filtered using standard quality control steps (Supplementary Figure 2, available online). GWAS testing for single SNP association was conducted using PLINK (11) (v1.9beta3), with the GFR decline after chemotherapy as the measure of toxicity and discretized cisplatin dosage as covariate with double-dose and normal-dose groups. The cutoff of 5 cycles was made to differentiate between normal and historically higher doses of cisplatin.

SNPs were annotated by ANNOVAR (v2015-06-17) (12) against the human reference genome hg19. Gene expression profiles were retrieved from GTExPortal (13).

We used a suggestive *P* value threshold of 1 × 10^−5^ (14) and a stringent threshold of 8.02 × 10^−8^ [Bonferroni corrected (15)].

In addition to the GWAS hits, 4 SNPs, rs11615 and rs3212986 (*ERCC1*), rs13181 (*ERCC2*), and rs316019 (*SLC22A2*), found in previous literature to be associated with cisplatin-induced nephrotoxicity (9), were added to the input feature search space in the machine learning modeling.

### Clinical Information

The clinical features used as input feature variables in the machine learning model were age at time of treatment, GFR before treatment, cumulative cisplatin dose per square meter of BSA, normal dose vs double-dose BEP, number of treatment cycles, histology (seminoma vs nonseminoma), prognostic classification as per IGCCCG (16) and stage of the disease as surrogate for size of retroperitoneal tumor size, which was represented as 3 features in the model (details on Supplementary Methods, available online).

### Statistical Analysis and Model Development

A random forest model (17), which identified different risk subgroups of GFR drop, was developed using SciKit-learn (18) in Python (v3.7.1). A GFR decline of more than 20% after chemotherapy was chosen as outcome to indicate a clinically significant change and to avoid selection of cases due to random variation. A 20% decline has been associated with, for example, cognitive deterioration (19) and risk of cardiovascular and all-cause mortality compared with those with stable GFR (20).

As a first stage, the predictive power of a model driven by clinical features only was established. In a second stage, genomic markers were added to the model.

From all 433 individuals, about 20% (78 individuals: 20 nephrotoxicity affected) of the data, with no missing values, was randomly separated ahead of time to be used as a holdout set. Therefore, for machine model training, we omitted those 78 individuals present on the holdout set and excluded individuals with missing data in either clinical or genomic data (Supplementary Figure 1, available online). Patients’ baseline characteristics in each of these sets are available in Supplementary Table 2 (available online).

Training and testing of the algorithm was performed with a 5 outer, 2 inner fold nested cross-validation (21,22) (Supplementary Figure 3, available online).

The sample-splitting process for training and testing cohorts was random and repeated 100 times. Area under the receiver operating characteristic curve (ROC-AUC) was used as the primary performance measure for model optimization.

**Figure 1.**
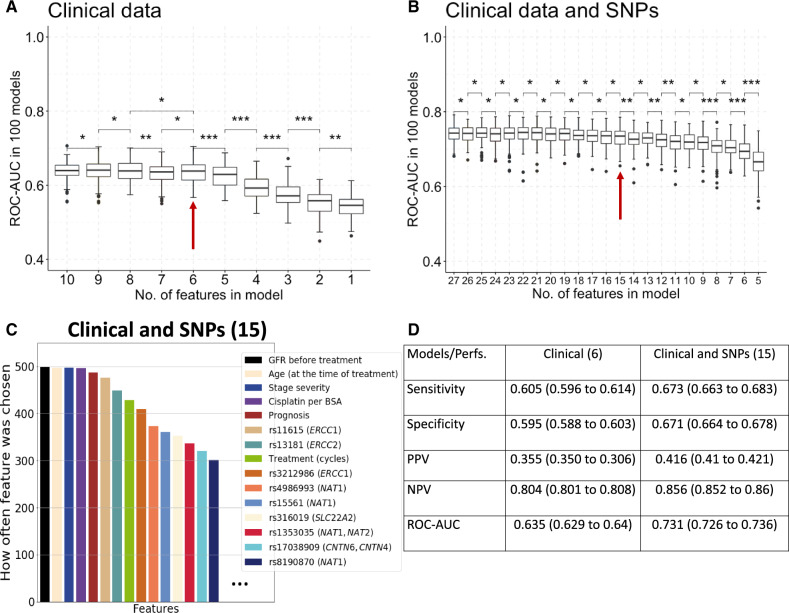
Feature selection using random forest-recursive feature elimination algorithm and diagnostic performances. **A** and **B**) Boxplots with different number of features, −10 to 1 and 27 to 5, for clinical and clinical plus genomics, respectively, and respective area under the receiver operating characteristic curve (ROC-AUC) throughout 100 different replications for data shuffling. **Asterisks** between boxplots represent *P* values (paired *t* test) of >.05 (*), ≤.05 (**), and ≤.01 (***). All tests were 2-sided. The **red arrow** represents the block chosen for further analysis. **C**) The features chosen the most on the 15-features clinical and SNP-based models. **D**) Performances obtained (mean and 95% confidence intervals) on the clinical models (6 features) and on the clinical and SNP-based models (15 features) using 0.50 cutoff for classification for sensitivity, specificity, positive predictive value, and negative predictive value. NPV = negative predictive value; Perfs. = performances; PPV = positive predictive value; ROC-AUC = area under the receiver operating characteristic curve; SNP = single-nucleotide polymorphism.

A recursive backwards feature elimination approach was used for feature selection initiated with 10 clinical features and then reduced (23). To identify when the algorithm should stop removing features, a paired *t* test (level of statistical significance, *P* < .05) was calculated for each round of feature elimination on mean ROC-AUCs (Figure 1, A and B). A statistically significant AUC drop (*P* < .05) was indicative of an important feature being eliminated. All statistical tests were 2-sided. Details on model optimization and variable importance are described in the Supplementary Methods (available online).

The top-ranked clinical features constituted the baseline for adding prioritized SNPs from GWAS (17 SNPs) and the literature (4 SNPs), and feature selection was done using recursive backwards feature elimination approach.

### Polygenic Risk Score (PRS)-Derived Models

We also calculated PRS-derived models weighted by effect sizes estimated by the GWAS using the R-Package PRSice (24). These were tested in the random forest models in place of individual SNPs. Two different approaches were used: the risks associated with all the 21 SNPs were combined to determine a PRS, and a PRS per gene was estimated.

### Model Performances and Risk Groups

The primary reported performance was assessed with a 0.50 cutoff on the random forest model scores. In addition, to determine clinical applicability, we assessed different cutoffs on the random forest scores with a goal of 10% false discovery or omission rate (positive or negative predictive values >90%).

For the SNPs and clinical-based models from the best round, the split that had a representative ROC-AUC close to the mean was used to assess different cutoffs (25) (Supplementary Figure 4, available online).

Based on this, specific cutoffs for detection of 3 risk groups were used on the holdout set: a high-risk group for developing nephrotoxicity; a low-risk group for developing nephrotoxicity; and an intermediate group, which refers to individuals whose prediction is not adequately compelling to change the clinical decision.

## Results

### Study Population

Overall, 433 individuals (26.8% nephrotoxicity affected) were assessed in this study, with a median (interquartile range [IQR]) age of 34 (27-43) years for affected patients (N = 116) and 30 years (26–37) for nonaffected patients (N = 317). The majority received 3 or 4 cycles of BEP. Before treatment, the median (IQR) GFR (mL/min/1.73 m^2^) was 128 (115-139) for affected and 119 (110-131) for nonaffected, and after treatment it decreased to 88 (75-99) for affected and 109 (100-119) for nonaffected (Table 1).

### Genome-Wide Association Study

Of 433 saliva samples received, 8 failed to yield high-quality genetic data. After quality control filtering, a total of 411 patients and 623 289 SNPs were eligible for GWAS (Supplementary Figures 1 and 2, available online).

**Figure 2.**
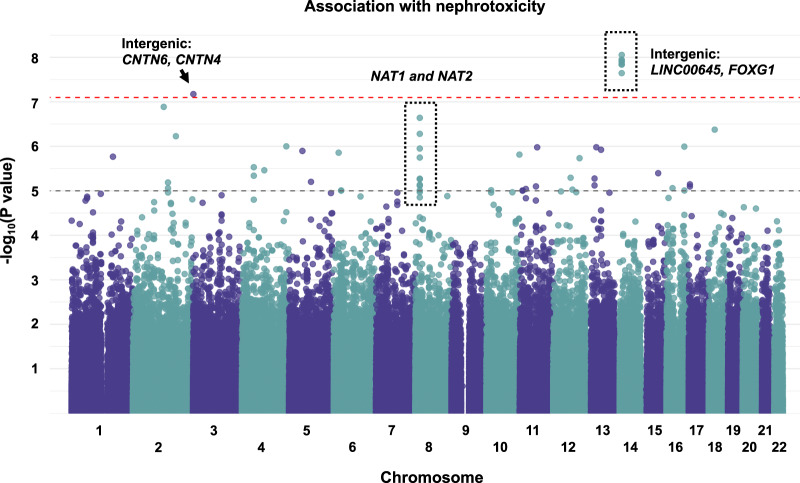
Genome-wide association study. Manhattan plot for association of 623 289 single-nucleotide polymorphisms with glomerular filtration rate decline. Linear model adjusted for cisplatin dosage was performed. The **black dashed line** represents a suggestive threshold: 1 × 10^−5^, and the **red dashed line** represents a stringent Bonferroni corrected threshold: 8.02 × 10^−8^. Markers in a contiguous pattern that pass the suggestive threshold are marked with a **dotted box.**

There was no indication of population stratification or inflation in the quantile-quantile plot of observed vs expected -log_10_ (*P* values) (Supplementary Figure 5, available online). GWAS controlling for cisplatin-based chemotherapy dosage identified 17 SNPs associated with GFR decline. Seven SNPs located contiguous on chromosome 14 within the intergenic region between *LINC00645* and *FOXG1* passed a genome-wide statistical significance threshold of *P* = 8.02 × 10^−8^ (Figure 2; Table 2). Nine additional SNPs located on chromosome 8, cytoband p22, passed a suggestive threshold of *P* = 1 × 10^−5^ and were located in the intron and 3´ untranslated region of *NAT1* or the intergenic region between *NAT1* and *NAT2*. SNP rs17038909 (*P* = 6.70 × 10^−8^), located in the intergenic region between *CNTN6* and *CNTN4*, passed the genome-wide statistical significance threshold.

These 17 SNPs were included in input feature space of the machine learning models.

### Risk Prediction Model

A baseline predictive model with only clinical features was trained using random forests. Of the initial 10 clinical features, 6 features were prioritized through recursive backwards elimination (Figure 1A): age at time of treatment, GFR before treatment, cumulative cisplatin-dose per square meter of BSA, number of treatment cycles, prognostic classification as per IGCCCG (1)^2^ (16), and stage of the disease, excluding group and histology. Univariate analysis also highlighted features selected in the random forest model (Table 1).

### SNPs and Clinical-Based Model

A selection of genomic markers was added to the baseline clinical prediction model: 17 SNPs from the GWAS and 4 additional SNPs from prior literature. Through recursive backwards elimination, 15 features were prioritized (6 clinical and 9 SNPs). The selected SNPs were rs11615 and rs3212986 (*ERCC1*), rs13181 (*ERCC2*), rs4986993, rs15561, rs8190870 (*NAT1*), rs1353035 (*NAT1/NAT2*), rs316019 (*SLC22A2*), and rs17038909 (*CNTN6/CNTN4*) (Figure 1, B and C). None of the SNPs located within the intergenic region between *LINC00645* and *FOXG1* were selected.

By adding genomic markers, ROC-AUC increased from 0.635 (95% confidence interval [CI] = 0.629 to 0.640) to 0.731 (95% CI = 0.726 to 0.736) (Figure 1D for additional performance metrics).

Additionally, 2 PRS were added independently to the baseline clinical model but did not outperform the individual SNPs (Supplementary Table 1, available online).

### Model Robustness

**Figure 3.**
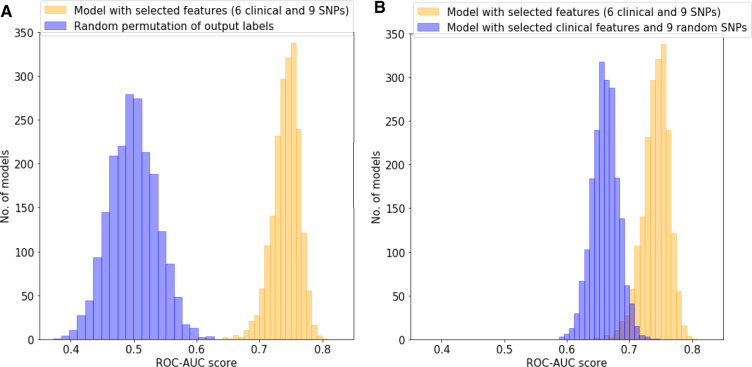
Benchmarking of the models. **A**) Test for random outcome simulated by permuting the labels 2000 times. **B**) Test for random single-nucleotide polymorphisms selection by combining 9 random markers, instead of the 9 selected markers, with the selected clinical traits. ROC-AUC = area under the receiver operating characteristic curve; SNP = single nucleotide polymorphism.

As a further validation, we tested for random outcome, simulated by permuting the labels 2000 times. This generated random performance for the model based on the clinical traits in combination with the 9 SNPs previously reported, with a ROC-AUC mean of 0.498 (95% CI = 0.497 to 0.500). Furthermore, to assess if the SNP selection was meaningful, the performance of 9 random GWAS SNPs instead of the previously described 9 selected SNPs was tested when combined with the selected clinical traits; this process was repeated 2000 times. This performed very similarly to clinical traits alone, with a ROC-AUC mean of 0.661 (95% CI = 0.660 to 0.661) against the model scores with a ROC-AUC mean of 0.742 (95% CI = 0.741 to 0.743) (Figure 3).

### Replication Dataset

**Figure 4.**
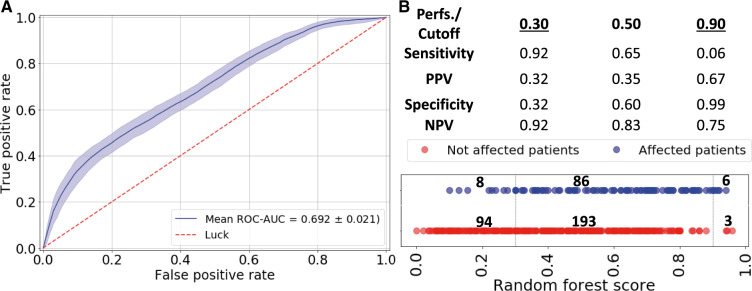
Final model evaluation (clinical and genomic markers) on the holdout set. **A**) Area under (AUC) the receiver operating characteristic curve (ROC; mean and 95% confidence interval) analysis of clinical risk factors and genetic variables for prediction of cisplatin-based nephrotoxicity in testicular cancer patients using the holdout dataset. **B**) Diagnostic performances obtained with 3 prediction cutoffs and independent evaluation (random forest score) for each individual: 78 individuals (×5 cross-validated models) (**blue**: affected; **red**: nonaffected). One validation external set was used. The 3 groups are represented: low-risk group (8% false negatives), undetermined zone, and high-risk group (33% false positives). Perfs. = performances; PPV = positive predictive value; NPV = negative predictive value; FN = false negatives; FP = false positives.

The holdout set (78 individuals: 20 nephrotoxicity affected) was used for replication of the random forest models with clinical and genetic features. A ROC-AUC of 0.692 (95% CI = 0.688 to 0.696) was obtained on the final evaluation (Figure 4A).

A prediction cutoff of 0.90 and 0.30 for high risk and low risk, respectively, of developing nephrotoxicity was chosen for further analysis on 1 validation external set to discuss the model clinical utility. A random forest score between 0.30 and 0.90 was not enough to make a clinical decision. In the high-risk group, we had a positive predictive value of 0.67 (33% false discovery rate) and specificity of 0.99 while capturing 6% of all nephrotoxicity, whereas in the low-risk group we had a sensitivity of 0.92 and negative predictive value of 0.92 (8% false omission rate), which captured 32% of all nonaffected patients (Figure 4B).

## Discussion

In this study, we were able to predict patients at risk of developing nephrotoxicity after BEP chemotherapy based on clinical and genetic features with a machine learning algorithm. Clinical features selected on the random forests–driven baseline clinical model were known risk factors of renal toxicity (2) and were statistically significant in univariate analysis. The aim of the baseline model was to mimic and codify clinical intuition, which relies on the available clinical information at the time of treatment.

When genomic markers were added to the baseline model, prediction power substantially improved. We believe that genomic information, although not being predictive on its own, improves a baseline clinical model for identification of patients at risk for nephrotoxicity.

PRS did not perform as well as independent SNPs when added to the model, suggesting that nonlinear correlations between SNPs drove the increase in performance opposed to the linear combination that PRS offer, as has also been suggested elsewhere (26).

SNPs located in the *LINC00645* and *FOXG1* intergenic regions, although strongly associated in the GWAS (*P* = 5 × 10^−8^), were not selected in the machine learning model because of either limited contribution or low minor allele frequencies (Table 2) that made it harder to detect in cross-validated setups.

**Table 2.** Top GWAS hits and literature SNP hits for cisplatin-based nephrotoxicity in testicular cancer patients^a^

SNP	Gene	CHR	Position	Region/Consequence	Alleles (ref/alt)	MAF (all)	MAF (EUR)	*P* ^b^
Top GWAS								
rs17038909	CNTN6, CNTN4	3	1467145	Intergenic	A/G	G: 0.10	G: 0.08	6.70 × 10^−8^
rs8190845	NAT1	8	18078628	Intronic	G/A	A: 0.20	A: 0.15	1.79 × 10^−6^
rs15561	NAT1	8	18080651	3 UTR	A/C	A: 0.44	A: 0.28	2.29 × 10^−7^
rs4986993	NAT1	8	18080747	3 UTR	T/G	T: 0.44	T: 0.28	5.25 × 10^−7^
rs8190870	NAT1	8	18081272	Downstream	C/T	T: 0.14	T: 0.15	1.12 × 10^−6^
rs13270034	NAT1, NAT2	8	18082354	Intergenic	G/A	A: 0.08	A: 0.13	7.64 × 10^−6^
rs13277177	NAT1, NAT2	8	18086096	Intergenic	A/G	G: 0.06	G: 0.10	9.72 × 10^−6^
rs13277481	NAT1, NAT2	8	18086217	Intergenic	A/G	G: 0.08	G: 0.13	5.47 × 10^−6^
rs13270961	NAT1, NAT2	8	18139163	Intergenic	T/C	C: 0.08	C: 0.11	7.31 × 10^-−6^
rs1353035	NAT1, NAT2	8	18140633	Intergenic	C/T	C: 0.15	C: 0.17	5.35 × 10^−6^
rs17095485	LINC00645, FOXG1	14	28500775	Intergenic	C/T	T: 0.07	T: 0.06	1.13 × 10^−8^
rs17382424	LINC00645, FOXG1	14	28529219	Intergenic	C/T	T: 0.02	T: 0.06	1.29 × 10^−8^
rs4551947	LINC00645, FOXG1	14	28584430	Intergenic	C/A	A: 0.05	A: 0.06	2.26 × 10^−8^
rs8020589	LINC00645, FOXG1	14	28604708	Intergenic	C/T	T: 0.07	T: 0.06	1.44 × 10^−8^
rs10131751	LINC00645, FOXG1	14	28681216	Intergenic	C/A	A: 0.07	A: 0.07	1.45 × 10^−8^
rs9671720	LINC00645, FOXG1	14	28714229	Intergenic	C/T	T: 0.05	T: 0.04	8.81 × 10^−9^
rs12323487	LINC00645, FOXG1	14	28837771	Intergenic	C/A/T	A: 0.09	A: 0.05	1.19 × 10^−8^
Literature								
rs316019	SLC22A2	6	160670282	Missense	A/C	A: 0.14	A: 0.11	0.21
rs13181	ERCC2	19	45854919	Stop gained	T/A/G	G: 0.24	G: 0.36	0.03
rs3212986	ERCC1	19	45912736	Stop gained	C/A/G/T	A: 0.30	A: 0.25	0.11
rs11615	ERCC1	19	45923653	Synonymous	A/G	A: 0.33	G: 0.38	0.004

^a^ Positions refer to assembly GRCh37. alt = alternative(s); CHR = chromosome; EUR = Europe; GWAS = genome-wide association study; MAF = minor allele frequency; ref = reference; ; SNP = single-nucleotide polymorphism; UTR = untranslated region.

^b^ A linear model was adjusted for cisplatin dosage and scored by *P* values representing how likely the variant association was by random chance.

SNPs rs4986993, rs15561, and rs8190870 *(NAT1*), rs1353035 (*NAT1/NAT2*), and rs17038909 (*CNTN6/CNTN4*) were newly discovered in the present GWAS to be associated with nephrotoxicity and added performance to the machine learning model.

*NAT1* and *NAT2* encode for arylamine *N*-acetyltransferases that take part in metabolizing drugs and chemical compounds in humans with a role in folate metabolism (27). These 2 genes encode similar protein sequences [identity = 81.03%, Clustal-Omega, Uniprot (28)], yet differ on expression profiles (13). *NAT1* is ubiquitously expressed in the central nervous system, and *NAT2* is specifically expressed in the liver, colon, and small intestine (Supplementary Figure 6, available online). It has been reported that cisplatin can impair NAT1 by blocking its transferase activity in human breast cancer cells and impair murine Nat2 activity in cultured mouse tissues (liver and kidney) (29), which on one hand contributes to the therapeutic effects of cisplatin, but on the other hand may lead to accumulation of cisplatin in the kidneys.

*CNTN6* and *CNTN4* encode for contacting proteins, which mediate cell surface interactions during nervous system development and have been suggested to be associated with neurodevelopmental disorders (30–32), though the association with nephrotoxicity needs to be further explored. SNPs found previously to be associated with nephrotoxicity were incorporated in this model. These SNPs were located at *ERCC1*, *ERCC2*, and *SLC22A2*.

*ERCC1* and *ERCC2* encode for excision repair proteins, and polymorphisms in *ERCC1*/*2* have been reported to alter ERCC1/2 DNA repair function (33–35), which may affect nephron repair capacity after cisplatin exposure during chemotherapy (36–39). If not adequately repaired, cisplatin-induced DNA damage can induce cell death (40,41).

*SLC22A2* encodes for organic cation transporter 2 (OCT2) protein, which is expressed in the proximal tubule epithelial cells of the kidney and involved in the absorption and excretion of xenobiotics and metabolites (42). OCT2 efficiently mediates cisplatin cellular uptake, leading to high cisplatin accumulation in renal proximal tubule cells (43) where cisplatin-induced nephrotoxicity typically occurs (44). OCT2 may be a key regulator in the renal accumulation of cisplatin, affecting drug handling and inducing nephrotoxicity (42,45).

During primary treatment of disseminated testicular cancer, about one-third of the patients develop cisplatin-induced nephrotoxicity (46,47).

This clinical and genomics-based model could be used as an early assessment for nephrotoxicity risk, assisting in identifying patients at high and low nephrotoxicity risk and influencing decisions on cisplatin chemotherapy cycles.

Using a 0.50 cutoff on the random forest model scores, we were able to achieve a sensitivity of 0.65, positive predictive value of 0.35, specificity of 0.60, and negative predictive value of 0.83. Differential thresholding of the nephrotoxicity model classified patients into high, low, and intermediate risk. For the high-risk group, the model correctly classified 67% of the patients who developed nephrotoxicity, yet only a small fraction of affected individuals was captured (0.06 sensitivity). On the other hand, for the low-risk group, the model correctly classified 92% of the patients who did not develop nephrotoxicity and captured 32% of the nonaffected population (Figure 4B).

Even though the model shows utility in the ability to predict toxicity throughout the score range, extreme cutoffs to identify the highest and lowest risk patients could point at the least disruptive implementation of such a model within current practice.

A strength of this study is the large dataset with a good representation of patients who developed nephrotoxicity after cisplatin-based chemotherapy, using exact renal measurements, and the first application, to our knowledge, of artificial intelligence on predicting such a phenotype.

The machine learning models appeared to be robust with stable performance across 100 random cross-validation splits of the training data, demonstrating performance of 0.731 mean ROC-AUC in cross-validation and 0.692 (95% CI = 0.688 to 0.696) ROC-AUC in the holdout set. Yet, as a limitation, the machine learning setups use some of the association results from the GWAS on the same cohort; therefore, replication on another cohort from an external dataset would be of substantial interest. *NAT1* and *NAT2* appear as interesting genetic targets to prioritize for assaying in future nephrotoxicity studies and would benefit from functional validation.

The ability to develop machine learning models for patient stratification in different nephrotoxicity risk groups has the potential to balance aggressive treatment against predicted toxicity risk.

In the future, toxicity may play a larger role in guiding treatment across several complex diseases, where data-driven prediction models may aid in decision making. Some of the clinical features used in this model, such as age at the time of treatment and GFR before chemotherapy as well as some of the identified genomics markers, could be applicable to other tumors types. Cisplatin is one of the most compelling drugs used in cancer treatment, and nephrotoxicity is a well-known side effect from its use. Our model could be applicable to ovarian, bladder, and lung cancer, where more elderly patients are at risk of nephrotoxicity and early identification of toxicity risks (or lack thereof) may influence treatment aggression or increase monitoring for selected patients.

## Funding

This work was supported by the Danish cancer society (R40-A2119). SLG was supported by Idella Foundation. ZZ and RLN were supported by Sino-Danish Center for Education and Research.

## Notes

**Role of the funder:** The funding source had no role in the design and conduct of the study; collection, management, analysis, and interpretation of the data; preparation, review, or approval of the manuscript; and decision to submit.

**Conflicts of interest:** RG is employed with Novo Nordisk Research Centre Oxford since February 2020. The other authors have no conflicts of interest to disclose.

**Author contributions:** **JL, GD, RG:** Study concept and design. **SLG, JL, ZZ, MB, RLN, RG:** Acquisition, analysis, or interpretation of data. **SLG, JL, ZZ:** Drafting of the manuscript. **SLG, JL, ZZ, MB, MDD, RLN, GD, RG:** Critical revision of the manuscript for important intellectual content. **SLG, ZZ:** Statistical Analysis. **MDD, GD, RG:** Study supervision.

## Supplementary Material

pkaa032_Supplementary_Data

## References

[1] Dilruba S, Kalayda GV. Platinum-based drugs: past, present and future. Cancer Chemother Pharmacol. 2016;77(6):1103–1124. 26886018 10.1007/s00280-016-2976-z

[2] Lauritsen J, Mortensen MS, Kier MGG, et al Renal impairment and late toxicity in germ-cell cancer survivors. Ann Oncol. 2015;26(1):173–178. 25361985 10.1093/annonc/mdu506

[3] Fung C, Fossa SD, Williams A, Travis LB. Long-term morbidity of testicular cancer treatment. Urol Clin North Am. 2015;42(3):393–408. 26216826 10.1016/j.ucl.2015.05.002

[4] Dasari S, Tchounwou PB. Cisplatin in cancer therapy: molecular mechanisms of action. Eur J Pharmacol. 2014;740:364–378. 25058905 10.1016/j.ejphar.2014.07.025 PMC4146684

[5] Astor BC, Hallan SI, Miller ER, Yeung E, Coresh J. Glomerular filtration rate, albuminuria, and risk of cardiovascular and all-cause mortality in the US population. Am J Epidemiol. 2008;167(10):1226–1234. 18385206 10.1093/aje/kwn033

[6] Karasawa T, Steyger PS. An integrated view of cisplatin-induced nephrotoxicity and ototoxicity. Toxicol Lett. 2015;237(3):219–227. 26101797 10.1016/j.toxlet.2015.06.012 PMC4516600

[7] Nematbakhsh M, Pezeshki Z, Eshraghi Jazi F, et al Cisplatin-induced nephrotoxicity; protective supplements and gender differences. Asian Pac J Cancer Prev. 2017;18(2):295–314. 28345324 10.22034/APJCP.2017.18.2.295 PMC5454720

[8] Achkar IW, Abdulrahman N, Al-Sulaiti H, Joseph JM, Uddin S, Mraiche F. Cisplatin based therapy: the role of the mitogen activated protein kinase signaling pathway. J Transl Med. 2018;16(1):96. 29642900 10.1186/s12967-018-1471-1 PMC5896132

[9] Zazuli Z, Vijverberg S, Slob E, et al Genetic variations and cisplatin nephrotoxicity: a systematic review. Front Pharmacol. 2018;9:1111. 30319427 10.3389/fphar.2018.01111 PMC6171472

[10] Kreiberg M, Bandak M, Lauritsen J, et al Cohort profile: The Danish Testicular Cancer late treatment effects cohort (DaTeCa-LATE). Front Oncol. 2018;8:37. 29515973 10.3389/fonc.2018.00037 PMC5826343

[11] Purcell S, Neale B, Todd-Brown K, et al PLINK: a tool set for whole-genome association and population-based linkage analyses. Am J Hum Genet. 2007;81(3):559–575. 17701901 10.1086/519795 PMC1950838

[12] Wang K, Li M, Hakonarson H. ANNOVAR: functional annotation of genetic variants from high-throughput sequencing data. Nucleic Acids Res. 2010;38(16):e164 20601685 10.1093/nar/gkq603 PMC2938201

[13] Lonsdale J, Thomas J, Salvatore M, et al The Genotype-Tissue Expression (GTEx) project. Nat Genet. 2013;45(6):580–585. 23715323 10.1038/ng.2653 PMC4010069

[14] MacArthur J, Bowler E, Cerezo M, et al The new NHGRI-EBI Catalog of published genome-wide association studies (GWAS Catalog). Nucleic Acids Res. 2017;45(D1):D896–D901. doi : 10.1093/nar/gkw1133 27899670 10.1093/nar/gkw1133 PMC5210590

[15] Bland JM, Altman DG. Multiple significance tests: The Bonferroni method. BMJ. 1995;310(6973):170–170. 7833759 10.1136/bmj.310.6973.170 PMC2548561

[16] International Germ Cell Cancer Collaborative Group. Germ cell consensus classification: a prognostic factor-based staging system for metastatic germ cell cancers. International Germ Cell Cancer Collaborative Group. J Clin Oncol. 1997;15(2):594–603. 9053482 10.1200/JCO.1997.15.2.594

[17] Breiman L. Random forests. Mach Learn. 2001;45(1):5–32.

[18] Pedregosa F, Varoquaux G, Gramfort A, et al Scikit-learn: Machine Learning in Python. *J Mach Learn Res.* 2011;12:2825–2830.

[19] Chen Y-C, Weng S-C, Liu J-S, Chuang H-L, Hsu C-C, Tarng D-C. Severe decline of estimated glomerular filtration rate associates with progressive cognitive deterioration in the elderly: a community-based cohort study. Sci Rep. 2017;7(1):42690. 28209982 10.1038/srep42690 PMC5314362

[20] Cheng T-Y, Wen S-F, Astor BC, Tao XG, Samet JM, Wen CP. Mortality risks for all causes and cardiovascular diseases and reduced GFR in a middle-aged working population in Taiwan. Am J Kidney Dis. 2008;52(6):1051–1060. 18706747 10.1053/j.ajkd.2008.05.030

[21] Picard RR, Berk KN. Data splitting. Am Stat. 1990;44(2):140–147.

[22] Varma S, Simon R. Bias in error estimation when using cross-validation for model selection. BMC Bioinformatics. 2006;7(1):91. 16504092 10.1186/1471-2105-7-91 PMC1397873

[23] Lai C, Guo S, Cheng L, Wang W. A comparative study of feature selection methods for the discriminative analysis of temporal lobe epilepsy. Front Neurol. 2017;8:633. 29375459 10.3389/fneur.2017.00633 PMC5770628

[24] Euesden J, Lewis CM, O’Reilly P. PRSice: Polygenic Risk Score software. Bioinformatics. 2015;31(9):1466–1468. 25550326 10.1093/bioinformatics/btu848 PMC4410663

[25] Parikh R, Mathai A, Parikh S, Chandra Sekhar G, Thomas R. Understanding and using sensitivity, specificity and predictive values. Indian J Ophthalmol. 2008;56(1):45–50. 18158403 10.4103/0301-4738.37595 PMC2636062

[26] Ho DSW, Schierding W, Wake M, Saffery R, O’Sullivan J. Machine learning SNP based prediction for precision medicine. Front Genet. 2019;10:267. 30972108 10.3389/fgene.2019.00267 PMC6445847

[27] Sim E, Abuhammad A, Ryan A. Arylamine N-acetyltransferases: from drug metabolism and pharmacogenetics to drug discovery. Br J Pharmacol. 2014;171(11):2705–2725. 24467436 10.1111/bph.12598 PMC4158862

[28] Consortium TU. UniProt: a worldwide hub of protein knowledge. Nucleic Acids Res. 2018;47(D1):D506–D515. 10.1093/nar/gky1049 PMC6323992 30395287

[29] Ragunathan N, Dairou J, Pluvinage B, et al Identification of the xenobiotic-metabolizing enzyme arylamine N-acetyltransferase 1 as a new target of cisplatin in breast cancer cells: molecular and cellular mechanisms of inhibition. Mol Pharmacol. 2008;73(6):1761–1768. 18310302 10.1124/mol.108.045328

[30] Hu J, Liao J, Sathanoori M, et al CNTN6 copy number variations in 14 patients: a possible candidate gene for neurodevelopmental and neuropsychiatric disorders. J Neurodev Disord. 2015;7(1):26. 26257835 10.1186/s11689-015-9122-9 PMC4528395

[31] Mercati O, Huguet G, Danckaert A, et al CNTN6 mutations are risk factors for abnormal auditory sensory perception in autism spectrum disorders. Mol Psychiatry. 2017;22(4):625–633. 27166760 10.1038/mp.2016.61 PMC5378808

[32] Tassano E, Uccella S, Giacomini T, et al Clinical and molecular characterization of two patients with CNTN6 copy number variations. Cytogenet Genome Res. 2018;156(3):144–149. 30508811 10.1159/000494152

[33] Ni M, Zhang W, Qiu J, et al Association of ERCC1 and ERCC2 polymorphisms with colorectal cancer risk in a Chinese population. Sci Rep. 2015;4(1):4112. 10.1038/srep04112 PMC3925949 24531312

[34] Yang L, Ritchie A-M, Melton DW. Disruption of DNA repair in cancer cells by ubiquitination of a destabilising dimerization domain of nucleotide excision repair protein ERCC1. Oncotarget. 2017;8(33):55246–55264. 28903417 10.18632/oncotarget.19422 PMC5589656

[35] Basu A, Krishnamurthy S. Cellular responses to cisplatin-induced DNA damage. J Nucleic Acids. 2010;2010:1–16. 10.4061/2010/201367 PMC2929606 20811617

[36] Khrunin A V, Moisseev A, Gorbunova V, Limborska S. Genetic polymorphisms and the efficacy and toxicity of cisplatin-based chemotherapy in ovarian cancer patients. Pharmacogenomics J. 2010;10(1):54–61. 19786980 10.1038/tpj.2009.45

[37] Tzvetkov MV, Behrens G, O’Brien VP, Hohloch K, Brockmöller J, Benöhr P. Pharmacogenetic analyses of cisplatin-induced nephrotoxicity indicate a renoprotective effect of ERCC1 polymorphisms. Pharmacogenomics. 2011;12(10):1417–1427. 21902499 10.2217/pgs.11.93

[38] Benhamou S, Sarasin A. ERCC2/XPD gene polymorphisms and cancer risk. Mutagenesis. 2002;17(6):463–469. 12435843 10.1093/mutage/17.6.463

[39] Windsor RE, Strauss SJ, Kallis C, Wood NE, Whelan JS. Germline genetic polymorphisms may influence chemotherapy response and disease outcome in osteosarcoma: a pilot study. Cancer. 2012;118(7):1856–1867. 21887680 10.1002/cncr.26472

[40] Zamble DB, Lippard SJ. Cisplatin and DNA repair in cancer chemotherapy. Trends Biochem Sci. 1995;20(10):435–439. 8533159 10.1016/s0968-0004(00)89095-7

[41] Rocha CRR, Silva MM, Quinet A, Cabral-Neto JB, Menck C. DNA repair pathways and cisplatin resistance: an intimate relationship. Clinics (Sao Paulo). 2018;73(suppl 1):e478s. 30208165 10.6061/clinics/2018/e478s PMC6113849

[42] Nigam SK, Wu W, Bush KT, Hoenig MP, Blantz RC, Bhatnagar V. Handling of drugs, metabolites, and uremic toxins by kidney proximal tubule drug transporters. Clin J Am Soc Nephrol. 2015;10(11):2039–2049. 26490509 10.2215/CJN.02440314 PMC4633783

[43] Ciarimboli G, Deuster D, Knief A, et al Organic cation transporter 2 mediates cisplatin-induced oto- and nephrotoxicity and is a target for protective interventions. Am J Pathol. 2010;176(3):1169–1180. 20110413 10.2353/ajpath.2010.090610 PMC2832140

[44] Leibbrandt ME, Wolfgang GH, Metz AL, Ozobia AA, Haskins JR. Critical subcellular targets of cisplatin and related platinum analogs in rat renal proximal tubule cells. Kidney Int. 1995;48(3):761–770. 7474662 10.1038/ki.1995.348

[45] Filipski KK, Loos WJ, Verweij J, Sparreboom A. Interaction of cisplatin with the human organic cation transporter 2. Clin Cancer Res. 2008;14(12):3875–3880. 18559608 10.1158/1078-0432.CCR-07-4793

[46] Prasaja Y, Sutandyo N, Andrajati R. Incidence of cisplatin-induced nephrotoxicity and associated factors among cancer patients in Indonesia. Asian Pac J Cancer Prev. 2015;16(3):1117–1122. 25735341 10.7314/apjcp.2015.16.3.1117

[47] Kidera Y, Kawakami H, Sakiyama T, et al Risk factors for cisplatin-induced nephrotoxicity and potential of magnesium supplementation for renal protection. PLoS One. 2014;9(7):e101902. 25020203 10.1371/journal.pone.0101902 PMC4096506

