# Supplementary material for: Prediction of Nephrotoxicity Associated With Cisplatin-Based Chemotherapy in Testicular Cancer Patients
Source: JNCI Cancer Spectr. 2020 Apr 23;4(3):pkaa032. doi: 10.1093/jncics/pkaa032 (PMC7315098; doi:10.1093/jncics/pkaa032)
Supplement: pkaa032_Supplementary_Data [file pkaa032_supplementary_data.pdf]

Supplementary Methods: Random forest structure

For random forest optimization, a tree search space of [1, 2, 3, 4, 5, 10, 50, 100, 250] was used in the inner-fold. Default values were used for the other hyperparameters, and can be assessed at SciKit-learn library v0.21.2 – RandomForestClassifier.

Splitting process for training and testing cohorts was replicated 100 times to generate an estimate of the variation of the area under the receiver operating characteristic curve (ROC-AUC) computed on the testing set. The mean ROC-AUC was then obtained from the 100 replication test sets. Throughout the feature selection, a set of features was chosen in each five outer fold, based on Gini importance, thus in each round of feature selection, we ended up with a total of 500 sets of features. For feature importance, we assessed which features were chosen the most. Feature selection is a critical step whose goal is to explore which features should be included to optimally predict an outcome and increase model generalizability, excluding redundant features. By using recursive backwards feature elimination coupled with random forest, the relevance of features is simultaneously assessed.

Ordinal categorical variables were encoded as one-column vectors, i.e. ({1,2,3}) for prognosis good, intermediate, and poor, respectively. Exception for stage severity feature, since we had information if the tumor was extragonadal, instead of germ cell tumor, there was one column with ({1,0}) for extragonadal or germ cell, respectively; one column representing the stage severity, ({1,2,3,4,5}), for stages I, IIa, IIb, IIc, III, respectively; and one column representing stage III with ({1,0}), in case the last one would contribute separately to the model and it would increase the predictive power in addition to stage severity. The nominal variable histology was encoded in one column, ({1,2}), for non-seminoma, or seminoma, respectively. Continuous variables were represented in absolute values. Single-nucleotide polymorphism data was encoded as one column vector with counts of minor alleles ({0,1,2}).

Supplementary Tables

Supplementary Table 1. Performances obtained with polygenic risk score*.

**Model**	**ROC-AUC (CI 95%)**
Clinical + PRS combined SNPs	0.671 (0.664-0.677)
Clinical + PRS per gene	0.697 (0.693-0.702)

*Area under the receiver operating characteristic curve on clinical and polygenic risk scores-based models using 100 replications for data shuffling.

Abbreviations: PRS, Polygenic risk score; SNP, single-nucleotide polymorphism; ROC-AUC, area under the receiver operating characteristic; CI, confidence interval.

Supplementary Table 2. Comparison of baseline characteristics between affected and non-affected patients (glomerular filtration rate high-drop).

		(A) Machine learning model with clinical data				(B) Machine learning model with clinical and genomics data				(C) Holdout set	
		Training		Testing		Training		Testing			
		Affected, No. (%)	Non-affected, No. (%)	Affected, No. (%)	Non-affected, No. (%)	Affected, No. (%)	Non-affected, No. (%)	Affected, No. (%)	Non-affected, No. (%)	Affected, No. (%)	Non-affected, No. (%)
**Number of patients**		36400 (50.0)	36400 (50.0)	9100 (26.8)	24800 (73.2)	31200 (50.0)	31200 (50.0)	7800 (25.7)	22500 (74.3)	20 (25.6)	58 (74.4)
**Age, median (IQR)**		34 (26-42)	30 (26-36)	34 (26-42)	30 (26-36)	35 (27-43)	30 (26-36)	35 (27-43)	30 (26-36)	34 (28-41)	32 (26-38)
**BEP regimen**	**Normal dose**	30000 (82.4)	34195 (93.9)	7500 (82.4)	23300 (94.0)	26400 (84.6)	29379 (94.2)	6600 (84.6)	21200 (94.2)	14 (70)	53 (91.4)
	**Double dose**	6400 (17.6)	2205 (6.1)	1600 (17.6)	1500 (6.0)	4800 (15.4)	1821 (5.8)	1200 (15.4)	1300 (5.8)	6 (30)	5 (8.6)
**GFR before treatment, median (IQR), mL/min/1.73m^2**		129 (115-140)	120 (110-132)	129 (115-140)	120 (110-132)	128 (112-139)	120 (110-132)	128 (112-139)	120 (110-132)	123 (115-141)	118 (109-127)
**GFR after treatment, median (IQR), mL/min/1.73m^2**		89 (75-99)	110 (101-120)	89 (75-99)	110 (101-120)	88 (75-100)	110 (101-120)	88 (75-100)	110 (101-120)	85 (74-98)	107 (97-115)
**Cisplatin, median (IQR), mg/m^2**		400 (390-400)	400 (300-400)	400 (390-400)	400 (300-400)	400 (386-400)	400 (300-400)	400 (386-400)	400 (300-400)	400 (397-638)	400 (300-400)
**Treatment cycles**	**3**	6800 (18.7)	10118 (27.8)	1700 (18.7)	7000 (28.2)	6400 (20.5)	8678 (27.8)	1600 (20.5)	6400 (28.4)	3 (15.0)	23 (39.7)
	**4**	23200 (63.7)	24077 (66.1)	5800 (63.7)	16300 (65.7)	20000 (64.1)	20701 (66.3)	5000 (64.1)	14800 (65.8)	11 (55.0)	30 (51.7)
	**5 or more**	1600 (4.4)	1487 (4.1)	400 (4.4)	1000 (4.0)	1200 (3.9)	1230 (3.9)	300 (3.9)	900 (4.0)	0 (0.0)	4 (6.9)
	**High-dose**	4800 (13.2)	718 (2.0)	1200 (13.2)	500 (2.0)	3600 (11.5)	591 (2.0)	900 (11.5)	400 (1.8)	6 (30.0)	1 (1.7)
**Histology**	**Seminoma**	8000 (22.0)	7331 (20.1)	2000 (22.0)	5000 (20.2)	7200 (23.1)	6752 (21.6)	1800 (23.1)	4900 (21.8)	1 (5.0)	17 (29.3)
	**Non-Seminoma**	28400 (78.0)	29069 (79.9)	7100 (78.0)	19800 (79.8)	24000 (76.9)	24448 (78.4)	6000 (76.9)	17600 (78.2)	19 (95.0)	41 (70.7)
**Prognostic group**	**Good**	22400 (61.5)	32168 (88.4)	5600 (61.5)	21900 (88.3)	19200 (61.5)	27962 (89.6)	4800 (61.5)	20100 (89.3)	12 (60.0)	49 (84.5)
	**Intermediate**	9600 (26.4)	3668 (10.1)	2400 (26.4)	2500 (10.1)	8000 (25.6)	2717 (8.7)	2000 (25.6)	2000 (8.9)	4 (20.0)	8 (13.8)
	**Poor**	4400 (12.1)	564 (1.5)	1100 (12.1)	400 (1.6)	4000 (12.8)	521 (1.7)	1000 (12.8)	400 (1.8)	4 (20.0)	1 (1.7)
**Stage**	**Extragonadal**	4400 (12.1)	2024 (5.6)	1100 (12.1)	1400 (5.6)	3200 (10.2)	1751 (5.6)	800 (10.2)	1300 (5.8)	2 (10.0)	0 (0.0)
	**Stage Im**	2400 (6.6)	3487 (9.6)	600 (6.6)	2300 (9.3)	2400 (7.7)	2951 (9.5)	600 (7.7)	2100 (9.3)	1 (5.0)	6 (10.3)
	**Stage IIa**	7600 (20.7)	9886 (27.1)	1900 (20.8)	6800 (27.4)	6400 (20.5)	8140 (26.1)	1600 (20.5)	5900 (26.2)	2 (10.0)	11 (19.0)
	**Stage IIb**	6000 (16.5)	8628 (23.7)	1500 (16.5)	5900 (23.8	5200 (16.7)	7366 (23.6)	1300 (16.7)	5300 (23.6)	6 (30.0)	18 (31.0)
	**Stage IIc**	7200 (19.8)	4390 (12.1)	1800 (19.8)	3000 (12.1)	6800 (21.8)	3932 (12.6)	1700 (21.8)	2800 (12.4)	4 (20.0)	11 (19.0)
	**Stage III**	8800 (24.2)	7985 (21.9)	2200 (24.2)	5400 (21.8)	7200 (23.1)	7060 (22.6)	1800 (23.1)	5100 (22.7)	5 (25.0)	12 (20.7)

(A) Machine learning model with clinical data: 339 individuals (training (down-sampling)/testing, 100

repetitions). **(B)** Machine learning model with clinical and genomics data: 303 individuals (training (down-sampling)/testing, 100 repetitions). **(C)** Holdout set: 78 individuals.

Abbreviations: No., number; IQR, interquartile range; BEP, bleomycin-etoposide-cisplatin; GFR, glomerular filtration rate.

Supplementary Figures

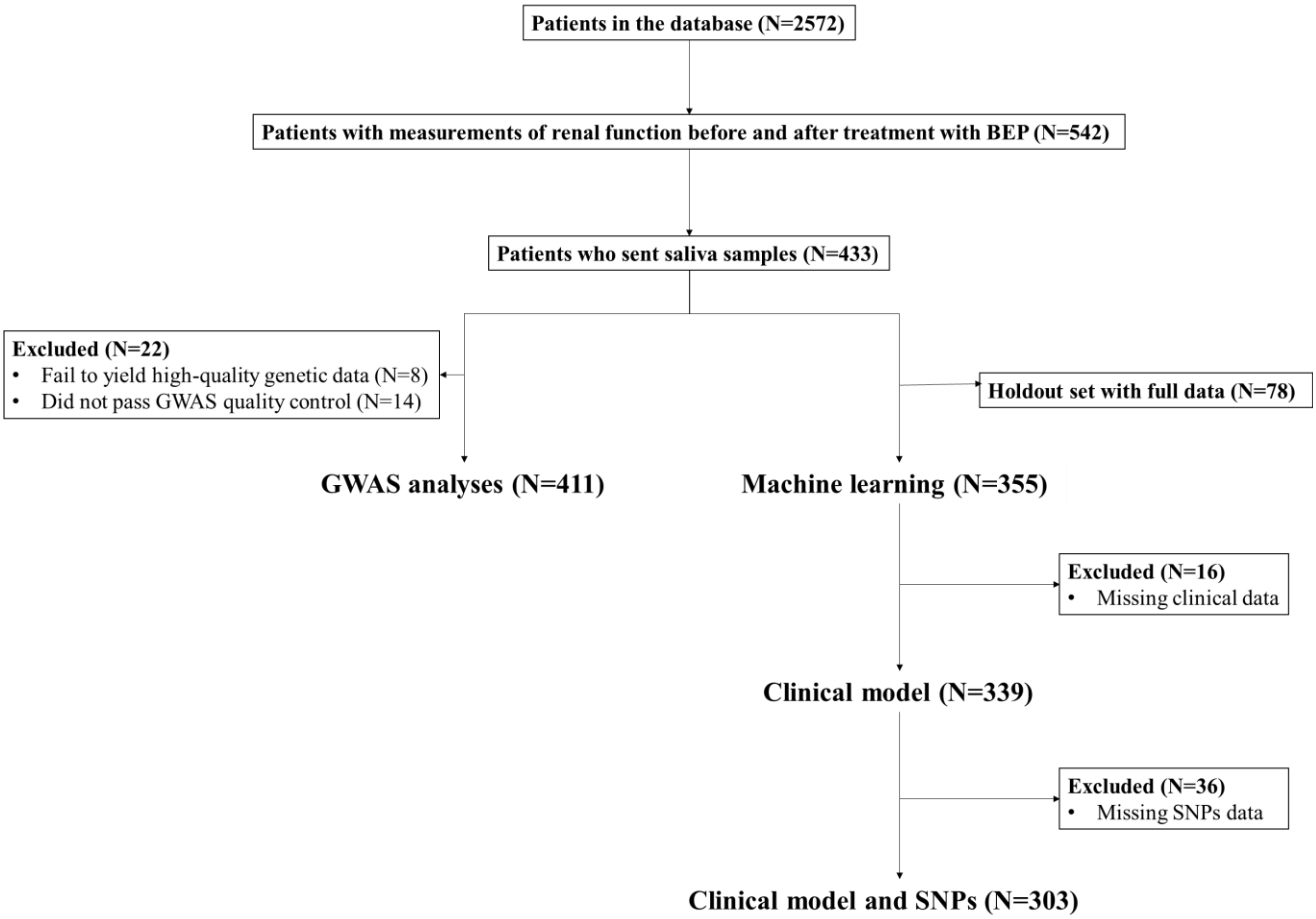

Supplementary Figure 1. Flowchart diagram. Abbreviations: N, number of patients; BEP, bleomycin-etoposide-cisplatin; GWAS, genome-wide association study; SNPs, single-nucleotide polymorphisms.

Step	Patients	SNPs
Step 0. Genotyping data retrieval<br>Data retrieval: 425 patients were genotyped for 964193 SNPs	425	964193
Step 1. Genotyping data preparation<br>Remove duplicated SNPs and those with ambiguous genome position, strand, and alleles	425	921861
Step 2. Removal of individuals/SNPs with low call rate<br>Keep individuals with call rate ≥ 96% and SNPs ≥ 98%	413	889050
Step 3. Removal of individuals with discordant sex information<br>Keep individuals with chromosome X homozygosity rate ≤ 20%	413	889050
Step 4. Removal of individuals with excessive heterozygosity rate<br>Keep individuals with heterozygosity rate ≤ 4 x S.D.	413	889050
Step 5. Removal of non-European individuals<br>Keep individuals clustered with European population from HapMap reference (≤ 4 x S.D. from cluster centroid mean)	412	889050
Step 6. Removal of related individuals<br>Keep individuals with identity-by-descent ≤ 0.1875	411	889050
Step 7. Removal of population outliers<br>Keep individuals within 4 x S.D. from the population structure cluster center mean	411	889050
Step 8. Removal of rare and non-HWE SNPs<br>Keep SNPs with MAF ≥ 1% and follow Hardy-Weinberg Equilibrium (p-value < 5 x 10^{-6})	411	623289

Supplementary Figure 2. Step-by-step demonstration of genomic data quality control. Abbreviations: S.D., standard deviation; MAF, minor allele frequency; SNPs, single-nucleotide polymorphisms; HWE, Hardy-Weinberg Equilibrium.

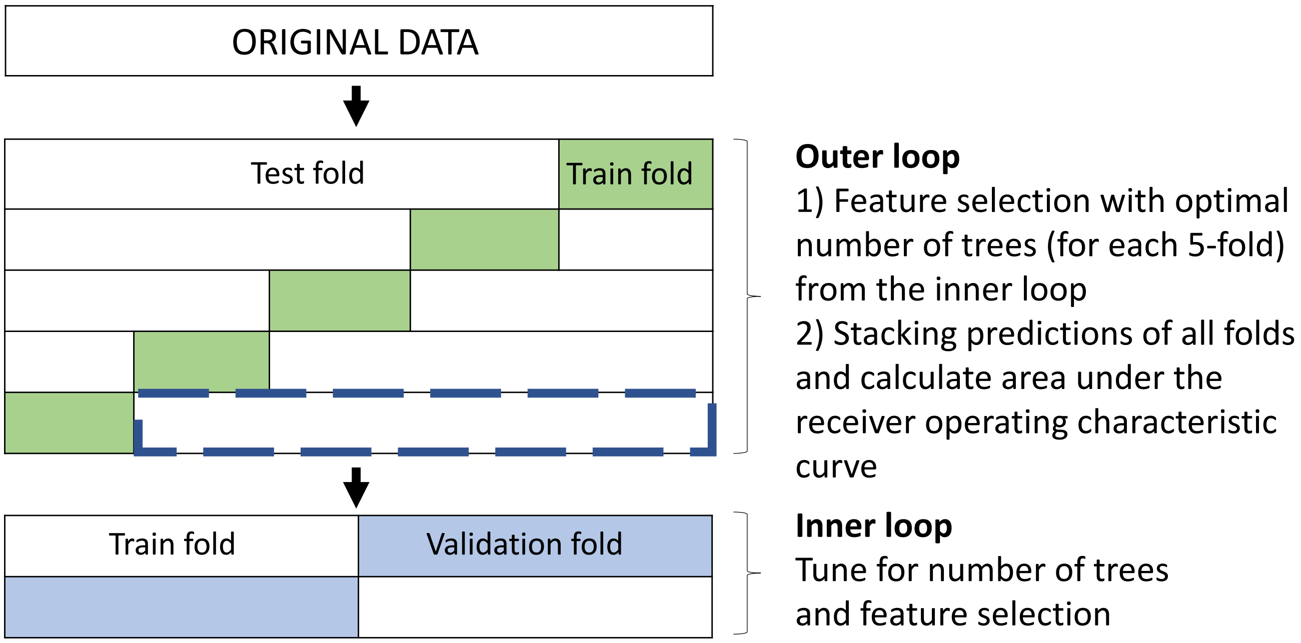

Supplementary Figure 3. Illustration of the five outer, two inner fold nested cross-validation used in this study.

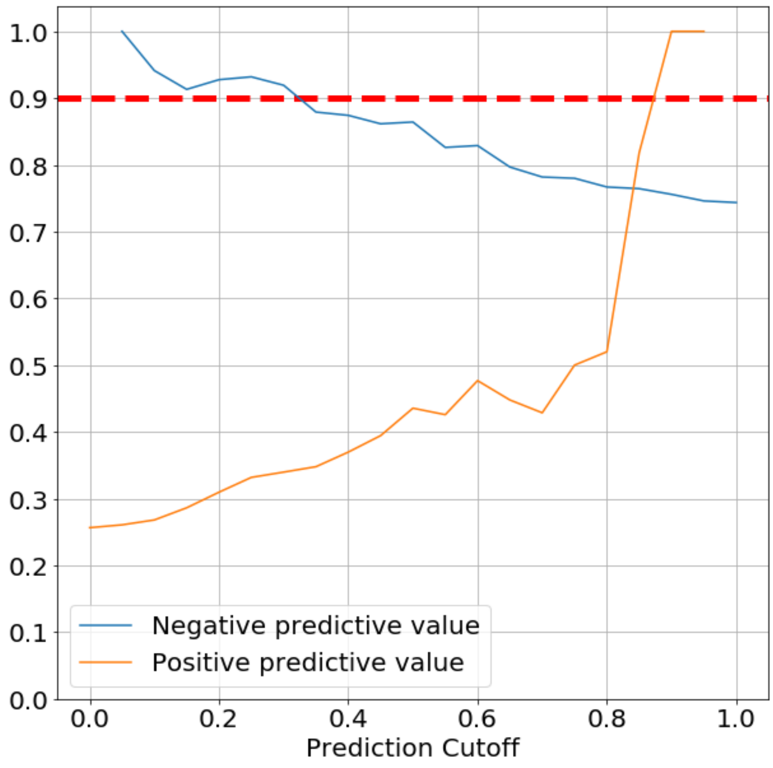

Supplementary Figure 4. Representation of performance changes on different cutoffs for negative and positive predictive value.

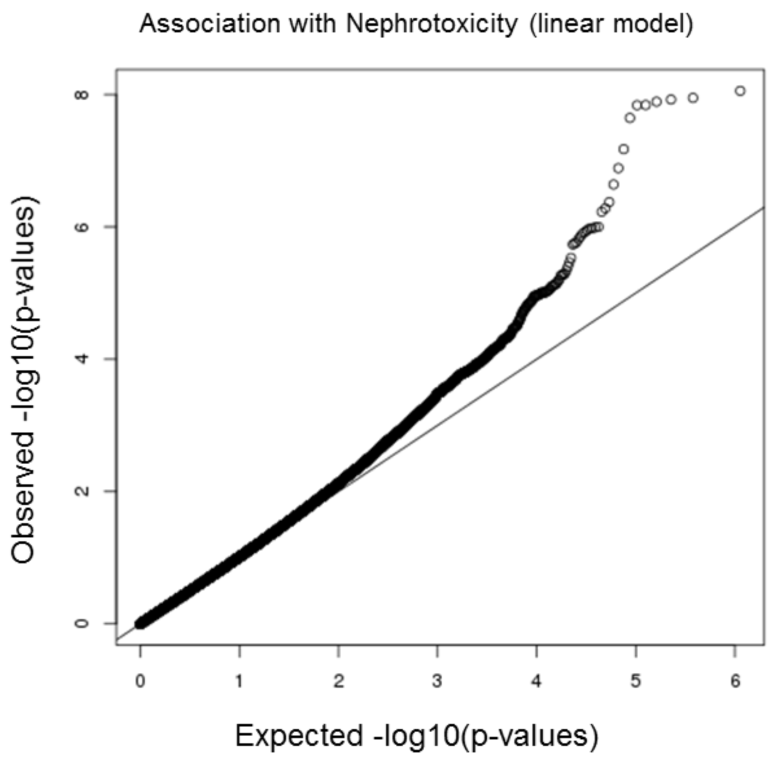

Supplementary Figure 5. Quantile-quantile plot of single-nucleotide polymorphisms p-values. Inflation factor (lambda) = 1.

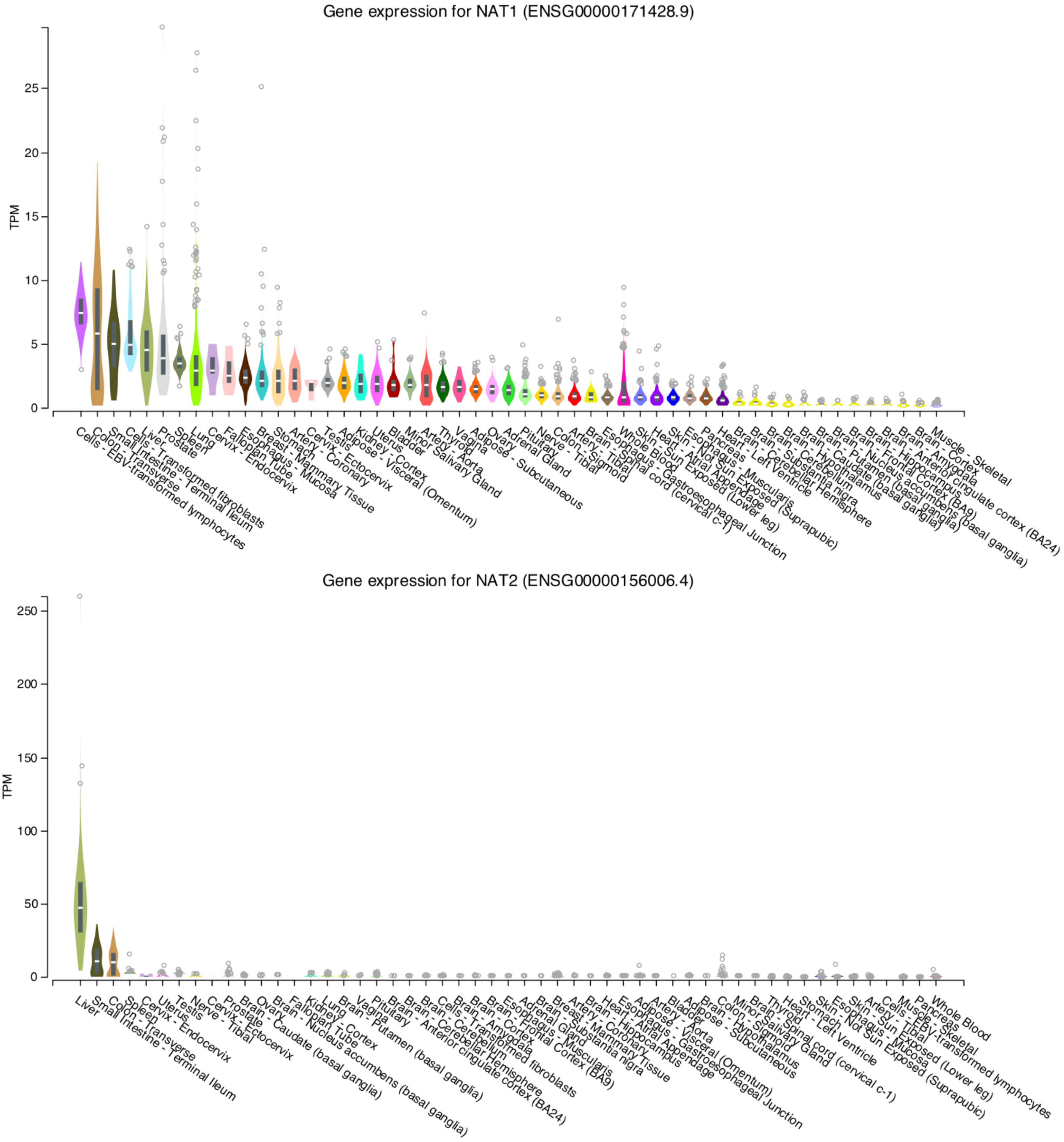

Supplementary Figure 6. Human body-wide expression profile of gene *NAT1* and *NAT2*. Data was retrieved from GTExPortal.
